# Change in treatment coverage and barriers to mental health care among adults with depression and alcohol use disorder: a repeat cross sectional community survey in Nepal

**DOI:** 10.1186/s12889-019-7663-7

**Published:** 2019-10-22

**Authors:** Nagendra P. Luitel, Emily C. Garman, Mark J. D. Jordans, Crick Lund

**Affiliations:** 1 Research Department, Transcultural Psychosocial Organization (TPO) Nepal, Kathmandu, Nepal; 2 Alan J Flisher Centre for Public Mental Health, Department of Psychiatry and Mental Health, University of Cape Town, Cape Town, South Africa; 3 Centre for Global Mental Health, Health Service and Population Research Department, Institute of Psychiatry, Psychology and Neuroscience, King’s College London, London, UK

**Keywords:** Mental health, Treatment coverage, Barriers to care, Stigma, Nepal

## Abstract

**Background:**

Despite the availability of evidence-based treatment, there is a substantial gap between the number of individuals in need of mental health care and those who receive treatment. The aim of this study was to assess changes in treatment coverage and barriers to mental health care among adults with depression and alcohol use disorder (AUD) before and after implementation of a district mental health care plan (MHCP) in Nepal.

**Methods:**

The repeat population-based cross-sectional community survey was conducted with randomly selected adults in the baseline (*N* = 1983) and the follow-up (*N* = 1499) surveys, 3 years and 6 months apart. The Patient Health Questionnaire and Alcohol Use Disorder Identification Test were used to screen people with probable depression and AUD. Barriers to seeking mental health care were assessed by using a standardized tool, the Barriers to Care Evaluation Scale (BACE).

**Results:**

The proportion of the participants receiving treatment for depression increased by 3.7 points (from 8.1% in the baseline to 11.8% in the follow-up) and for AUD by 5.2 points (from 5.1% in the baseline to 10.3% in the follow-up study), however, these changes were not statistically significant. There was no significant reduction in the overall BACE score in both unadjusted and adjusted models for both depression and AUD. The possible reasons for non-significant changes in treatment coverage and barriers to care could be that (i) the method of repeat population level surveys with a random sample was too distal to the intervention to be able to register a change and (ii) the study was underpowered to detect such changes.

**Conclusion:**

The study found non-significant trends for improvements in treatment coverage and barriers to mental health care following implementation of the district mental health care plan. The key areas for improvement in the current strategy to improve treatment coverage and barriers to mental health care included change in the content of the existing community sensitization program, particularly for changing attitude and intention of people with mental illness for seeking care.

## Background

Globally, mental, neurological and substance use (MNS) disorders are among the leading causes of disability, contributing to 10.4% of global disability adjusted life years (DALYs) [1]. MNS disorders are also considered as significant risk factors contributing to pre-mature deaths [2], and often result in adverse social and economic consequences [3]. Among the MNS disorders, depression and alcohol use disorder (AUD) are reported to be the second and third leading causes of years lived with disability [4–6]. While, there is an increasing evidence base of cost-effective interventions for mental health problems, it is reported that more than half (56%) of people with depression [7] and 87% people with alcohol abuse and dependence do not receive any treatment [8]. The most common factors hindering mental health care utilization included low perceived needs, stigma and discrimination associated with mental illness, lack of awareness about the available services, inability to afford the treatment cost and lack of effective treatment [9–14].

In Nepal, few studies have been conducted in the area of mental health. Most of the prior studies have focused on estimating prevalence of mental health problems, particularly the mental health problems of populations affected by conflict and other humanitarian crises. The available data shows a wide range of reported prevalence of depression (14.0 to 80.0%), anxiety (22.9 to 81.0%), and posttraumatic stress disorder (PTSD) (3.0% to 60.0) [15–17]. Few studies have attempted to estimate treatment coverage for mental health care among individuals suffering from such disorders. A recent study conducted among adults in Chitwan district (southern Nepal), however, reported a very large treatment gap for depression (91.5%) and alcohol use disorder (94.9%) [18]. The most commonly reported barriers to treatment were inability to afford care, fear of being perceived as weak for having mental health problems, fear of being perceived as crazy and being too unwell to ask for support [14].

Over the past decade, several initiatives have been taken globally to minimize the treatment gap for mental health problems. The PRogramme for Improving mental health care (PRIME), a research program consortium aims to minimize the enormous treatment gap on mental health care by generating new evidence on implementation and scaling up of mental health programs in primary and maternal health settings in low and middle-income countries (LMICs) [19]. As part of PRIME, a district mental health care plan (MHCP) was developed and implemented in Chitwan. The MHCP consists of intervention packages delivered at community, health facility and health organization platforms [20]. The evaluation of the PRIME district mental health care plan was carried out using multiple methods which included measuring change in population level treatment contact coverage; change in detection and initiation of evidence-based treatment, and change in health and socio-economic outcomes of people receiving treatment from primary health clinics [21, 22]. A community survey was conducted before and 3 years after implementation of the PRIME MHCP to assess the changes in population-level contact coverage and barriers to seek mental health services. The overall aim of this paper is to report on the change in treatment coverage and barriers to mental health care among adults with depression and alcohol use disorder before and 3 years after implementation of PRIME in Chitwan Nepal.

## Methods

### Setting

Nepal is one of the poorest countries in south Asia, and has a total population of approximately 26.4 million with 69.1 years life expectancy at birth. Nepal’s gross national income per capita at purchasing power parity (PPP) was $2500 in 2017, ranking 193, out of 226 countries [23]. The study was conducted in Chitwan, a district in southern Nepal. The total population of Chitwan district is 579,984 (279,087 male and 300,897 female), with approximately 132,462 households. On average, 4.38 people live in each household in the district. The literacy rate of Chitwan is 78.9%, which is higher than the national average of 67% [24]. Although mental health services are restricted to a few hospitals located in big cities in Nepal; in Chitwan, mental health services (both inpatient and outpatient services) are available in the district hospital and private medical colleges operating in the district.

### Study design

We used quantitative methods for data collection in both baseline and the follow-up survey. We used a repeat population-based cross-sectional survey design to assess the change in treatment contact coverage and barriers to mental health care among adults with depression and alcohol use disorder. The baseline community survey was conducted between May and July 2013 (before the implementation of the PRIME MHCP) and the follow-up community survey was conducted between December 2016 and February 2017 (3 years after the start of the implementation of the PRIME MHCP).

### Sampling and sample size

Two different samples were recruited for the baseline and follow-up surveys. Sample size was calculated to allow detection of a change in contact coverage between the baseline and the follow-up study with 80% statistical power and two-sided alpha of 0.05 [18]. The estimated contact coverage for depression and AUD in the baseline was between 0 to 5%, and hypothesized to increase to between 20 to 30% at the post-MHCP. The estimated sample size for both baseline and follow-up surveys was 1500.

Participants were recruited from 10 Village Development Committee (VDCs) of Chitwan district. VDCs are the lowest administrative units in a district covering a population size of 5000 to 25,000. Households were used as the sampling unit for the surveys, and the same multi-stage random sampling technique was used to recruit participants at baseline and in the follow-up study. First, the total sample size was divided into 90 wards (9-wards in each VDC based on the proportion of the total population of each ward. Second, the required numbers of households from each ward were selected using a systematic random sampling technique. For this purpose, we prepared a list of all households (with the name of head of households) for each of the 90 wards. We calculated a sampling frame for each ward using the proposed sample size and total households of a particular ward. At the end, we selected the required number of households by using the calculated sampling frame. Finally, the research assistants selected one adult from each household by using simple random selection procedure. The field workers first prepared a list (roster) of all the members living in each household. A member of each household drew a name of one eligible participant from within that household. If no one was found at the household after three visits, or the selected adult was not willing to participate in the study then the research assistant visited the nearest neighbouring household to assess its members for the inclusion criteria. In total, we recruited 1983 and 1499 adults in the baseline and the follow-up study, respectively.

### Participants and procedure

The inclusion criteria were age 18 years or above, resident of the study VDCs, ability to provide informed consent and fluency in the Nepali language. The exclusion criteria included having severe mental illness and unable to provide informed consent. Twelve Nepali-speaking research assistants with an undergraduate degree were hired for data collection. Research assistants visited each sampled household, assessed eligibility criteria, performed sampling procedures within the household, and obtained informed consent from the selected participants for the interview. Interviews were conducted in the respondents’ place of residence by using Android tablets with questionnaire application. The research assistants provided information about the survey in both oral and written format prior to the recruitment of the participants. The selected literate adults then signed the consent form to participate in the study. The study was approved by Nepal Health Research Council (NHRC), the national ethical body of the government of Nepal; ethical review board of World Health Organization (WHO) Geneva, and University of Cape Town.

### Instruments

Standardized and validated instruments were used to screen people with depression and AUD and to assess barriers to mental health care. We have described each of the study measures in detail below.

#### Demographic characteristics

Basic socio-demographic characteristics of the respondents such as age, sex, education, caste/ethnicity, marital status, religion, occupation, and family income were collected for each of the study participants in both baseline and follow-up study.

#### Patient health questionnaire (PHQ9)

The PHQ9 was used to screen people with depression. PHQ9 is a widely used self-report screening tool for patients with depression in various medical settings [25]. The PHQ9 has nine common symptoms of depression and respondents are asked to score those symptoms based on their experiences in the past 2 weeks. The PHQ9 has been translated and validated in Nepal [26]. The validated cut off score of ≥10 (sensitivity =0.94, specificity =0.80) has been recommended for moderate to severe depression symptoms [26]. In addition to the PHQ9, we also asked an additional question to assess depressive episodes in the past 12-months period. We considered those with an affirmative response to the additional question or a score of 10 or more on the PHQ9 to have depressive disorder.

#### Alcohol use disorder identification test (AUDIT)

The AUDIT has been used to screen people with alcohol abuse or dependence. The AUDIT is a 10 item tool developed by the World Health Organization (WHO) to assess alcohol consumption, drinking behaviours, and alcohol related problems among people presenting with current symptoms or symptoms over the past 1 year [27]. AUDIT has been translated, adapted and validated in Nepal. A cut off score of 9 or more has been recommended for alcohol dependence or alcohol abuse for both males (sensitivity 0.97 and specificity 0.92) and females (sensitivity 0.94 and specificity 0.91) [28].

#### Barriers to access to care evaluation (BACE)

Barriers related to stigma and discrimination and other non-stigma related barriers were assessed using the BACE scale, which was developed by involving both experts and service users at Kings College London [29]. The BACE is a 30-item self-report instrument where respondents are asked whether each of the items has ever stopped, delayed or discouraged them for receiving or continuing care for their mental health problems. It has a four-point response scale ranging from 0 (not at all) to 3 (a lot) along with ‘66’ for non-applicable responses. The total score of BACE ranges from 0 to 90; a higher score indicates more barriers. We followed a systematic approach that has been developed in Nepal for translation and adaptation of standardized tool for translation and contextualization of BACE in Nepal [30].

#### Treatment contact coverage

Respondents who had reported depressive episodes in the past 12 months or a score of 10 or more on the PHQ9 or score of 9 or more on the AUDIT were subsequently asked whether they had sought treatment for that disorder in the past 1 year. Based on the framework described by Tanahashi [31], contact coverage was defined as the proportion of individuals with depression or AUD who accessed any health care providers for that condition in the past 12 months. Health care providers were disaggregated into mental health specialists, generalists, primary health care workers and other community-based care providers.

### Statistical analysis

Data were transferred from the online data collection application to Stata version-13, where data were cleaned and analyzed. Participant data was weighted according to the inverse probability of sampling (i.e. 1 / (probability of selecting a household within the ward X probability of selecting an adult within the household). First, we described the demographic and screening-related characteristics of the participants who were recruited into the baseline and follow-up survey. As all socio-demographic variables were categorical, we presented numbers and proportions, and used Chi-square tests to compare demographic characteristics in the baseline and follow-up survey. We used logistic regression to assess if the change in the proportion of the participants who accessed mental health care (treatment contact coverage) differed between the baseline and follow-up survey. This was conducted separately for participants with depression and AUD. In a separate analysis (data are not presented in this manuscript), we found that there was no association between socio-demographic characteristics and help-seeking behavior in the baseline data, and the sample size was also too small to adjust potential confounders. Hence, we presented unadjusted risk ratio (RR), 95% confidence interval (CI) and *P* value.

To assess changes in barriers to seeking mental health care from baseline to follow-up, we compared the overall scores on the BACE scale, and scores on BACE sub-scales (i.e. stigma, financial barriers, cultural beliefs and practices, low perceived needs, perceived ineffectiveness of available services, lack of support, and lack of knowledge) between baseline and follow-up surveys. Given the skew in the distribution of the overall score on BACE scale and scores on BACE sub-scales, we used negative binominal regression analysis, and presented both unadjusted and adjusted incidence rates ratio (IRR), 95% confidence interval (CI) and *P* value separately for depression and AUD. Models were adjusted for socio-demographic variables which were significantly different from baseline to follow-up. Finally, logistic regression analysis was performed to assess the association between ‘help seeking’ behaviour and barriers to mental health care in the baseline survey. It is likely that there are factors that may influence both barriers and help-seeking, but these were not measured in the study. Only demographic variables were measured, and as reported above none of the demographic variable were associated with help-seekign at baseline. Therefore, we presented unadjusted odds ratio (RR), 95 confidence interval (CI) and *P* values. As the number of people receiving treatment for depression and AUD was relatively small, both disorders have been combined for regression analysis.

## Results

Table 1 presents socio-demographic characteristics of the participants involved in the baseline and the follow-up surveys. The proportion of female participants in the follow up survey (*n* = 1072, 68.3%) was greater than that at baseline (*n* = 1280, 60.1%; χ2 = 18.3, *p* < 0.001). More than two-thirds of the sample in both baseline (*n* = 1418, 68.1%) and follow-up survey (*n* = 1089, 69.5%) were of working age (25 to 59 years); married (baseline, *n* = 1645, 81.5% and follow up, *n* = 1253, 82.8%) and Brahmin/Chhetri (baseline, *n* = 948; 48.3% and follow up, *n* = 772, 51.5%). A large majority of the participants in the follow-up survey (*n* = 1351; 90.4%) were from the households with sufficient family income for foods for 9 to 12 months; this proportion was significantly greater than that at baseline (*n* = 1324; 67.8%, χ2 = 126.0, *p* < 0.001). The prevalence of depression in the follow-up survey (*n* = 118; 7.6%) was significantly lower than that found at baseline (*n* = 228; 11.1%, χ2 = 9.3, *p* = 0.002).

**Table 1:** Socio-demographic characteristics of the participants in the baseline and follow-up surveys

Variables	Baseline (*N* = 1983)		Follow-up (*N* = 1499)		χ2, p
	*N*	^a^ %	*N* ^a^	%	
*Sex*					
Male	703	39.9	427	31.7	18.3, < 0.001
Female	1280	60.1	1072	68.3	
*Age (years)*					
18–24	296	18.4	221	17.1	0.43, 0.649
25–59	1418	68.1	1089	69.5	
60 and above	269	13.5	189	13.3	
*Education*					
Not schooling	275	13.2	176	11.8	7.5, < 0.001
Literate/less than primary	315	14.9	304	19.9	
Primary	360	17.6	381	22.7	
Secondary	822	41.6	518	36.1	
College /University	211	12.7	120	9.5	
*Marital status*					
Single	215	13.6	135	10.7	3.6, 0.027
Married	1645	81.5	1253	82.8	
Others (widow/divorced/separated)	123	4.9	111	6.5	
*Caste/Ethnicity*					
Brahmin/Chhetri	948	48.3	772	51.5	1.0, 0.391
Janajati	542	27.4	388	25.3	
Dalit	308	15.0	229	13.7	
Others	185	9.3	110	9.5	
*Religion*					
Hindu	1604	80.3	1239	82.4	1.6, 0.201
Non-Hindu	379	19.7	260	17.6	
*Occupation*					
Agriculture	1335	64.2	839	55.6	16.3, < 0.001
Service/Business	297	15.5	204	13.5	
Students/Unemployed	244	15.0	384	26.2	
Others	107	5.3	72	4.8	
*Family income sufficient to manage foods for the period of*					
Up to 6 months	352	16.8	29	1.7	126.0, < 0.001
6 to 9 months	307	15.4	119	7.9	
9–12 months or above	1324	67.8	1351	90.4	
*Clinical characteristics*					
Screen positive on PHQ-9	228	11.1	118	7.6	9.3, 0.002
Screen positive on AUDIT	96	5.0	74	4.9	0.04, 0.839

^a^ %, sample weighted percent;

*N* non-weighted sample size

### Treatment coverage

Table 2 presents percentages of the participants who had sought treatment from a specialist, generalist, or other health care providers for symptoms related to depression and alcohol use disorder in the last one-year period. Some of these results have already been published elsewhere [22]. Of the total 118 participants with depression in the follow-up survey, 11.8% (*n* = 13) reported that they had received treatment from any providers in the past 12 months; this proportion was greater but not significantly different from the proportion reported at baseline (*n* = 18; 8.1%; RR = 1.40, *p* = 0.336). Similarly, the proportion of the participants receiving treatment for AUD from any providers in the follow-up survey (*n* = 9; 10.3%) was greater than, but not significantly different from, that found at baseline (*n* = 5; 5.1%; RR = 2.33, *p* = 0.115). There were no significant differences in the proportions of the participants receiving treatment from either mental health specialists or generalists health workers (e.g. medical doctors, health assistants) for both depression and AUD between baseline and follow-up surveys (Table 2).

**Table 2:** Help-seeking behavior of people with depression or alcohol use disorder in the baseline and follow-up surveys

	Depression				AUD^b^			
*Types of providers*	Baseline (*N* = 228) n (%)^a^	Follow-up (*N* = 118) n (%)	Unadjusted Risk Ratio (95% CI)	*P*-value	Baseline *N* = 96) n (%)	Follow-up (*N* = 74) n (%)	Unadjusted Risk Ratio (95% CI)	*P*-value
Receiving treatment in the past year from any providers (Follow-up)	18 (8.5)	13 (11.8)	1.44 (0.68–3.06)	0.337	5 (5.1)	9 (10.3)	2.52 (0.81–7.87)	0.112
*Type of service providers*								
Generalists (e.g. Doctors and PHC workers) (Follow-up)	5 (1.8)	4 (4.2)	1.106 (0.28–3.94)	0.933	2 (1.3)	3 (3.2)	3.97 (0.41–38.25)	0.232
Mental health specialists (e.g. psychiatrists, psychologists) (Follow-up)	9 (3.6)	8 (5.6)	1.18 (0.45–3.05)	0.738	0	1 (1.6)	–	–
Others (Traditional healers, religious leaders) (Follow-up)	8 (4.2)	5 (5.2)	0.73 (0.25–2.19)	0.580	4 (4.5)	3 (3.6)	0.99 (0.22–4.44)	0.992

*n* non-weighted frequency

^a^ *%, sample weighted percent;*

^b^ includes both men and women

### Change in perceived barriers for mental health care

The changes in the overall BACE score and scores on BACE sub-scales (i.e. stigma, financial barriers, cultural beliefs and practices, low perceived needs, perceived ineffectiveness of available services, lack of support, and lack of knowledge) between baseline and follow-up surveys are presented in Table 3. The changes in the overall BACE scores were not significant in both unadjusted and adjusted regression models for both depression and AUD. The results of the unadjusted modules showed a significant reduction in financial barriers (IRR = 0.73, CI, 0.56–0.96, and *P* = 0.025) and lack of support (IRR = 0.72, CI, 0.55–0.95 and *P* = 0.021) among the participants with depression. Once adjusted, however, the reduction was marginal for both financial (IRR = 0.80 and *P* = 0.135) and lack of support (IRR = 0.78 and *P* = 0.112). For the AUD groups, the reduction was significant only in the financial sub-scale (IRR = 0.68, CI, 0.47–0.99 and *P* = 0.044) in the unadjusted model (Table 3).

**Table 3:** BACE overall and subscale scores in the baseline and follow-up surveys

	Mean (SD)		Unadjusted		Adjusted	
BACE overall and subscales (Number of items)	Baseline	Follow-up	IRR^a^ (CI)	P	IRR^a^ (CI)	P
*Depression*						
Overall BACE (30)	34.0 (13.0)	29.4 (12.3)	0.87 (0.68–1.00)	0.239	0.92 (0.71–1.20)	0.557
Stigma (12)	14.3 (6.3)	12.2 (6.7)	0.85 (0.67–1.09)	0.202	0.92 (0.70–1.20)	0.542
Financial barriers (3)	3.8 (1.8)	2.8 (1.6)	0.73 (0.56–0.96)	0.025	0.80 (0.59–1.07)	0.135
Cultural practices and beliefs (4)	3.1 (1.6)	2.8 (1.5)	0.88 (0.67–1.16)	0.376	0.92 (0.68–1.24)	0.583
Low perceived needs (4)	4.4 (2.3)	4.6 (2.1)	1.04 (0.81–1.35)	0.738	1.13 (0.85–1.50)	0.405
Lack of knowledge about available services (1)	1.3 (0.91)	1.5 (0.91)	1.18 (0.87–1.60)	0.275	1.20 (0.84–1.69)	0.296
Perceived ineffectiveness of services (3)	2.4 (1.7)	2.1 (1.6)	0.87 (0.65–1.50)	0.324	0.89 (0.65–1.22)	0.462
Lack of support (3)	3.3 (2.0)	2.4 (1.5)	0.72 (0.55–0.95)	0.021	0.78 (0.58–1.06)	0.112
*AUD*						
Overall BACE (30)	31.2 (13.2)	25.5 (13.5)	0.82 (0.59–1.13)	0.223	0.78 (0.54–1.11)	0.172
Stigma (12)	13.0 (7.2)	10.6 (6.6)	0.82 (0.59–1.14)	0.232	0.75 (0.52–1.08)	0.127
Financial barriers (3)	3.5 (1.7)	2.4 (1.7)	0.68 (0.47–0.99)	0.044	0.70 (0.46–1.06)	0.095
Cultural practice and beliefs (4)	3.0 (1.6)	2.6 (1.5)	0.86 (0.59–1.25)	0.437	0.87 (0.57–1.31)	0.507
Low perceived needs (4)	4.0 (2.2)	3.9 (2.3)	0.97 (0.68–1.38)	0.850	0.90 (0.61–1.32)	0.587
Lack of knowledge about available services (1)	1.4 (1.0)	1.3 (1.0)	0.93 (0.61–1.41)	0.720	0.86 (0.54–1.37)	0.529
Perceived ineffectiveness of services (3)	2.1 (1.6)	1.9 (1.6)	0.91 (0.62–1.34)	0.632	0.87 (0.75–1.35)	0.547
Lack of support (3)	2.7 (1.7)	2.8 (8.1)	1.02 (0.71–1.49)	0.839	0.83 (0.40–1.29)	0.415

^a^ *IRR* Incidence risk ratio

### Factors associated with help seeking behavior and barriers for mental health care

The association between help seeking behaviours of people with depression or alcohol use disorder and barriers for mental health care are presented in Table 4. Results show that the financial barrier is significantly associated with help-seeking behavior at baseline: participants reporting more financial barrier had lower odds of receiving mental health treatment in the 12 months preceding the survey (OR = 0.76; CI = 0.59–0.99; *p* = 0.042). Likewise, participants experiencing greater lack of support were less likely (OR = 0.78 and CI = 0.61–1.01) to receive care than their counterparts who reported greater support, though the difference was marginal (*p* = 0.063) (Table 4).

**Table 4:** Association between help seeking behaviour and barriers for mental health care in the baseline survey

	Received treatment in the past 12 months		Unadjusted Odds ratio (95% CI)	*P* value
Barriers to mental health care	No [Mean (SD)]	Yes [Mean (SD)]		
Overall BACE	32.1 (13.3)	27.6 (10.6)	0.97 (0.94–1.00)	0.111
Stigma	13.6 (6.6)	11.6 (6.5)	0.96 (0.98–1.02)	0.167
Financial barriers	3.6 (1.8)	2.8 (1.2)	0.76 (0.59–0.99)	0.042
Low perceived needs	4.2 (2.3)	4.0 (2.2)	0.96 (0.80–1.15)	0.665
Cultural practice and beliefs	3.0 (1.6)	2.4 (1.2)	0.78 (0.58–1.04)	0.095
Lack of support	3.1 (1.9)	2.3 (1.5)	0.78 (0.61–1.01)	0.063
Lack of knowledge about available services	1.3 (0.96)	1.1 (1.0)	0.81 (0.52–1.27)	0.362
Perceived ineffectiveness of services	2.3 (1.7)	2.0 (1.5)	0.90 (0.70–1.17)	0.448

## Discussion

This study assessed changes in treatment coverage and barriers to mental health care among people with depression and AUD in Chitwan, Nepal. The study revealed a very large treatment gap in both baseline and the follow-up survey for depression (91.9% at baseline and 88.2% at follow-up) and AUD (94.9% at baseline and 89.7% at follow-up). The proportion of the participants receiving treatment for both depression and AUD increased at the follow-up survey but the changes were not statistically significant. Due to lack of sufficient data on population level contact coverage in the context of interventions, possibilities for comparison are limited. The treatment contact coverage for depression reported in the follow-up survey was much smaller than that found in the cross-sectional studies in the LMICs. For example, studies conducted in 10 LMICs as a part of WHO world mental health survey initiatives reported that 52.6% of people with a need for depression care had contacted any service provider in the past 12 months [32]. Similarly, the reported contact coverage for depression in the follow-up study was also smaller than that found in nationally representative studies in South Africa (15.3%) [33], Central India (23.5%), Ethiopia (23.7%) [18] and Northern India (21%) [34]. However, the treatment contact coverage reported in the follow-up survey was larger than that found in China (3.4%) [35], Korea (6.1%) [36]; Nigeria (1.6%); Colombia (5.5%) and Ukraine (7.2%). Similarly, the treatment contact coverage reported for AUD in the follow-up survey was smaller than that found in Ethiopia (13.1%) [37]; however, this was larger than that found in central India (2.8%), Uganda (3.5%) [18] and South Africa [33]. Few studies have investigated the change in treatment contact coverage on mental health care in the LMICs. The available studies demonstrated mixed findings on the effectiveness of community mental health programs in increasing treatment contact coverage. For example, a study conducted to evaluate an integrated mental health program in India and Pakistan showed a significant improvement in the treatment contact coverage on mental health care in India [38]; however, the results were not promising in Pakistan [38]. The treatment contact coverage for depression increased 6-times (i.e. 4.3% in the baseline to 27.2% in the endline) in an 18-month interval community survey conducted as part of VISHRAM (Vidarbha Stress and Health Programme) project in central India [39].

Despite the efforts made by PRIME to sensitize the general community on mental health issues, increased availability of mental health services in the health facilities, engagement of FCHVs on detection and referral of people with mental illness and anti-stigma programs, the proportion of the participant receiving services reported in the follow-up survey is smaller than anticipated. This is especially the case given that the community informant detection tool (CIDT), which has shown to increase help seeking behaviour among people in the same community in Chitwan [40], was used to facilitate detection of people with probable mental health problems in the community. Similarly, the ability of trained primary health care workers to detect mental health problems in the health facilities also increased significantly after the introduction of the mhGAP-based training program [22]. The possible reasons for not achieving a significant changes in the treatment contact coverage could be explained by the small number of people screened positive for depression and AUD, that was not sufficient to detect population level change in treatment contact coverage. Another important possible explanation for non-significant improvements in the treatment coverage could be explained by the distal nature of the outcome in relation to the intervention. This is borne out by the fact that the district MHCP did show a significant increase in the number of people utilizing services over time [22].

Results also demonstrated that perceived barriers to mental health care did not change over time, after controlling for demographic differences between participants at baseline and follow up. The possible reasons for this could be that there were no targeted community-level activities. Most of our community level activities targeted increasing mental health literacy, and making people aware about the services available in the project sites. Studies have demonstrated that mental health literacy can help to change attitude but the evidence that it leads to help-seeking behavior is largely lacking [41]. Still, studies have demonstrated that attitude and intentions can predict behavior [42], therefore, the content of the existing community sensitization program need to be revised and additional content, particularly for changing attitude and intention of people towards seeking mental health care included. Our results contrast with the study conducted in Andra Pradesh, India, where they found significant improvements in stigma related to help-seeking behaviours after implementation of the community level awareness programs [43]. Our unadjusted results are consistent with the study conducted in Rawalpindi, Pakistan and Bangalore, India, where a significant reduction was reported in financial barriers 3 months after implementation of community based mental health programs [38].

The findings of this study may have several implications to improve access to mental health services through primary and community health care systems in Nepal. First, the results demonstrated that the proportions of the participants receiving mental health services increased at the follow-up survey after introducing the evidence-based treatment program in primary and community health care system, although this change was not statistically significant. Considering the huge population-wide coverage of primary health care services in Nepal, integration of mental health services into primary and community health care system could be a potential strategy to reduce the alarming treatment gap in mental health care in Nepal. Second, despite the efforts made at the community level to minimize barriers to mental health care, the results demonstrated that there was no significant change in most of the barriers in both depression and AUD groups in the follow-up survey. The content included in the existing community sensitization program may not have been sufficient to change attitude and intention of people with mental illness about the needs, and effectiveness of the available mental health services. Therefore, the content of the existing community sensitization program may need to be revised and additional contents targeting to change the attitude and intention of people with mental illness for seeking care included. A third implication of our study is towards improving infrastructure and quality of the available services in primary care. In general, most of the primary health care facilities in the study sites lack separate and confidential rooms for consultations. Due to stigma and discrimination associated with mental illness, people generally do not want to share their problems in front of other people. This has also been supported by the proportion of the participants reporting a high level perceived stigma in the follow-up survey. Moreover, lack of confidential places in the primary health care facilities was reported to be an important barrier for improving demand side barriers [44], as well as one of the important system level barriers for integration of mental health into primary care [45]. Therefore, a separate and confidential place should be made available in each health facilities for consultation. Finally, we assessed the barriers by using the BACE scale which has attempted to include various barriers that are relevant for people with depression and AUD; however, more and other barriers, potentially non-assessed barriers might need to be overcome to further increase greater help-seeking behavior. Therefore, further research, mainly a qualitative study, is recommended to investigate why people with depression and AUD do not seek services even though services are made available free of cost in their own community.

This study has limitations that may have impacted our comparisons pre- and post-service initiation. First, we found a low proportion of male participants in both baseline and the follow-up survey, which could be explained by a high out-migration of the adult male population in the study areas. The recent census recorded an absent population of 7.3% i.e. 1,921,494, of which 87.6% were male and 12.4% were female [46]. Similarly, a significantly larger proportion of participants in the follow-up survey were from the households with sufficient family income for foods for 9 to 12 months compared to baseline. This might have impacted on the difference identified in help seeking behaviours of the participants from baseline to follow-up. Second, the prevalence of people screening positive for depression and AUD in both surveys was relatively lower than anticipated [18], and so we had less than 80% statistical power to detect a 20% change in treatment-seeking. Third, the PHQ9 which was used to screen people for depression has approximately 4–6 false positive per 10 patients screening positive for depression with fewer than one per 100 false negatives while using a cut-off score of 10 or above with 0.94 sensitivity and 0.80 specificity [26]. Fifth, due to the lack of data on exposure to PRIME community level intervention, we cannot conclude that the change reported in the treatment contract coverage was due to PRIME interventions. Finally, despite the thorough sampling procedure and analysis of weighted data, the sample recruited may still not be representative of the district due to diverse population and small geographical coverage.

## Conclusion

This is, to the best of our knowledge, the first study conducted to assess changes in treatment coverage and barriers to mental health care in Nepal for people with probable depression and AUD. The study found a non-significant trend for improvements in population level treatment coverage following implementation of the district mental health care plan. Furthermore, the results also demonstrated non-significant reduction in most of the barriers to mental health care before and after implementation of the district mental health care plan. The possible reasons for non-significant changes in treatment coverage and barriers to care could be that the method of repeat population level surveys with a random sample was too distal to the intervention to be able to register a change and that the study may have been underpowered to detect such changes. The key areas for improvement in the current strategy to improve treatment coverage and barriers to mental health care included change in the content of the existing community sensitization program, particularly for changing attitude and intention of people with mental illness for seeking care.

## Data Availability

Interested parties may notify the PRIME investigators of their interest in collaboration, including access to the data set analyzed here, through the following website: http://www.prime.uct.ac.za/contact-prime.

## Abbreviations

AUD: Alcohol use disorder
AUDIT: Alcohol use disorder identification test
BACE: Barrier to care evaluation scale
CIDT: Community informant detection tool
FCHV: Female community health volunteers
LMIC: Low and middle income countries
MHCP: Mental health care plan
NMS: Mental, neurological and substance use disorder
PHC: Primary health care
PHQ: Patients health questionnaire
PRIME: Programme for improving mental health care
VDC: Village development committee
WHO: World health organization
